# Supplementary material for: Effectiveness of a school-based high-intensity interval training intervention in adolescents: study protocol of the PRO-HIIT cluster randomised controlled trial
Source: Front Pediatr. 2024 Oct 29;12:1458610. doi: 10.3389/fped.2024.1458610 (PMC11554476; doi:10.3389/fped.2024.1458610)
Supplement: Supplementary file 1 [file Datasheet1.pdf]

*Visual 2-back task*

Working memory will be assessed using a modified visual 2-back task (26). Stimuli are sequences of animals (i.e., pig, giraffe, panda, snail and duck), which will be presented on the screen one-by-one. Participants will be instructed to respond to the stimulus as quickly and accurately as possible by either pressing the "F" button on the keyboard if the stimulus is identical to the one that appeared two trials back or "J" if not. The task consists of 52 trials in total, with a 1-minute rest when participants have finished the first 26 trials. Among them, 16 (one third) are target trials and 32 are foil trials. Whether the current trial is a target or foil is randomly decided when programming the task. A trial starts with a blank screen for 500 ms, followed by the stimulus for 1500 ms and another blank screen for 1000 ms. Responses within 200 and 2500 ms after stimulus onset are considered valid. The task will start with a few sentences of instruction (e.g., *You will see a list of animal images shown on the screen one by one. Press "F" if the animal matches the one 2 images back, otherwise, press "J"*). This will be followed by an 8-trial practice task (two target trials), with more than 2 wrong answers deemed as a failure in the practice stage. Participants will be given another chance to pass the practice and move to the main tasks or otherwise, they will be moved directly to the next stage of the test.

*Flanker task*

Participants' inhibition will be assessed using a modified Flanker task (26). In the task, participants will respond as quickly and accurately as possible to the direction of the fish in the middle which is flanked by another four fishes that are either swimming towards the same direction (congruent) or opposite direction (incongruent) to the central target fish. Participants will be instructed to press the "F" button on the keyboard if the fish swims towards left or "J" if towards right. The task consists of 104 trials in total, with a 1-minute rest when participants have finished the first 52 trials. The task contains four trial types: congruent left, congruent right, incongruent left and incongruent right, which will be equally and randomly distributed in the first and second half of the task. A trial starts with a blank screen for 500 ms, followed by the stimulus for 333 ms and another blank screen for 1067 ms. Responses within 200 and 1400 ms after stimulus onset are considered valid. The task will start with a few sentences of instruction (e.g., *The fishes are swimming to either the left or right. You should pay attention to the fish in the middle. Press 'F' if the fish is swimming left. Press 'J' if the fish is swimming right.*). This will be followed by an 8-trial practice task (two trial types each in a random order), with more than 2 wrong answers deemed as a failure in the practice task. Participants will be given another chance to pass the practice and move to the main tasks or otherwise they will be moved directly to the next stage of the test.

*Colour-shape switch task*

Cognitive flexibility will be evaluated by a modified version of colour-shape switch task (26). In the task, participants will be instructed to react as quickly and accurately as possible to press the "F" button or "J" on the keyboard, with the following cues: *If the object is at the top of the screen: press "F" if it is blue, press "J" if it is green; If the object is at the bottom of the screen: press "F" if it is square, press "J" if it is triangular*. Participants will be provided with a 24-trial practice task (16 homogeneous trials including a block of 8 colour only and a block of 8 shape only trials, and 8 heterogeneous trials which switch from 4 colour and 4 shape trials at random). Participants need to make at least 18 valid responses to move to the real trial, however, they will be provided with two chances to pass the practice and likewise, they will be moved to the next stage if they fail in both practices. The task contains eight trial types: top (blue square and triangular, purple square and triangular) and bottom (blue square

and triangular, purple square and triangular), which will be equally and randomly distributed in the first and second half of the task. A trial starts with a blank screen for 500 ms, followed by the stimulus for 5000 ms. Responses within 200 and 5000 ms after stimulus onset are considered valid. The task consists of 128 trials in total, with a 1-minute rest when participants have finished the first 64 trials.
